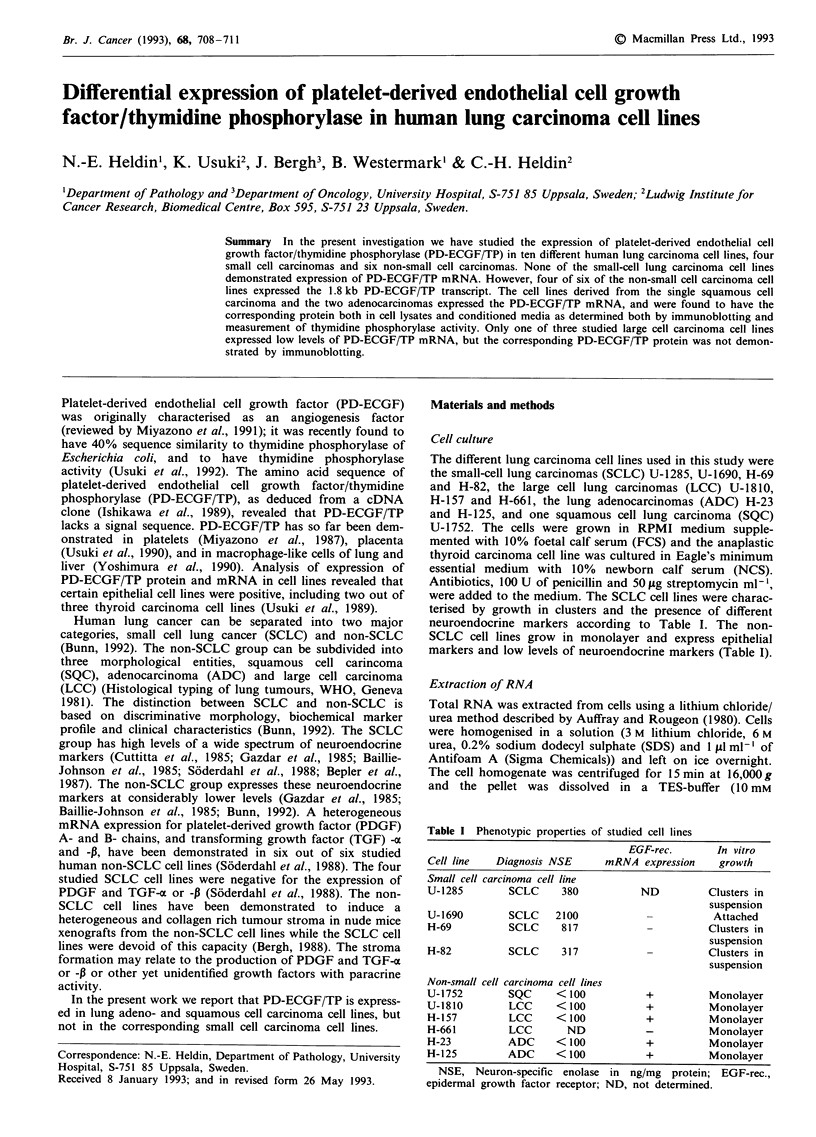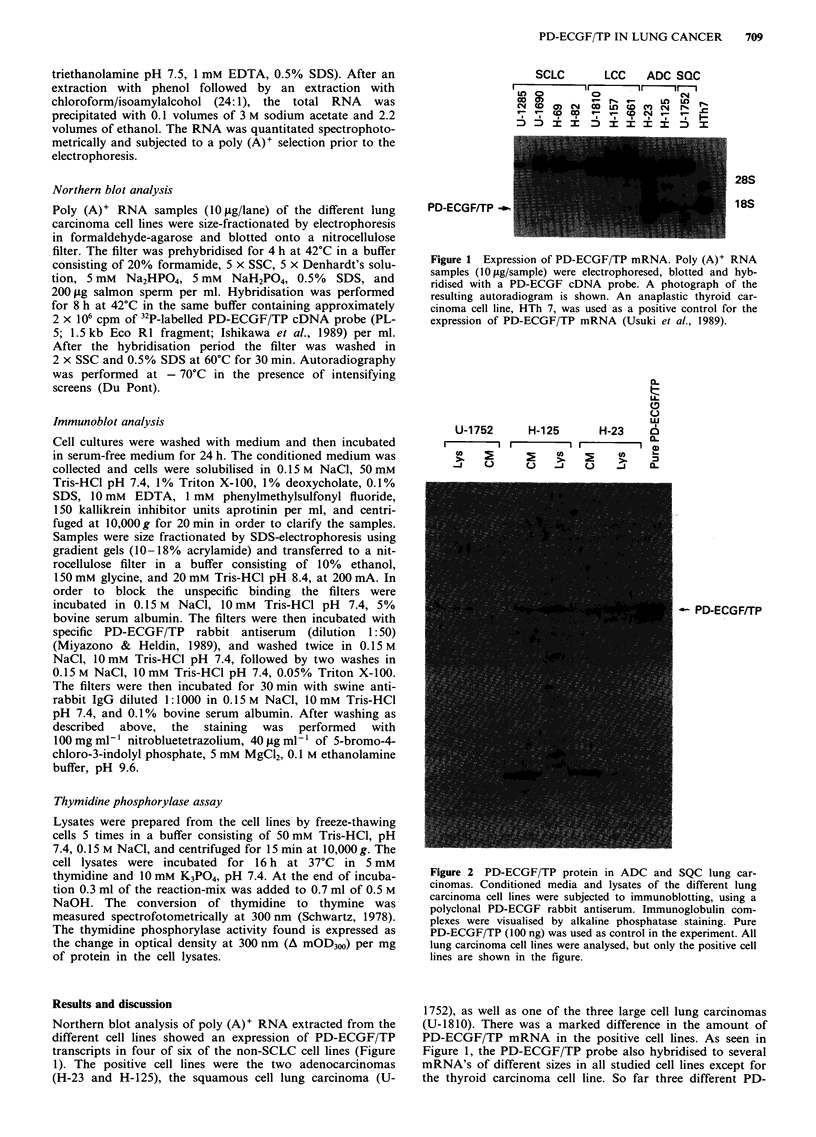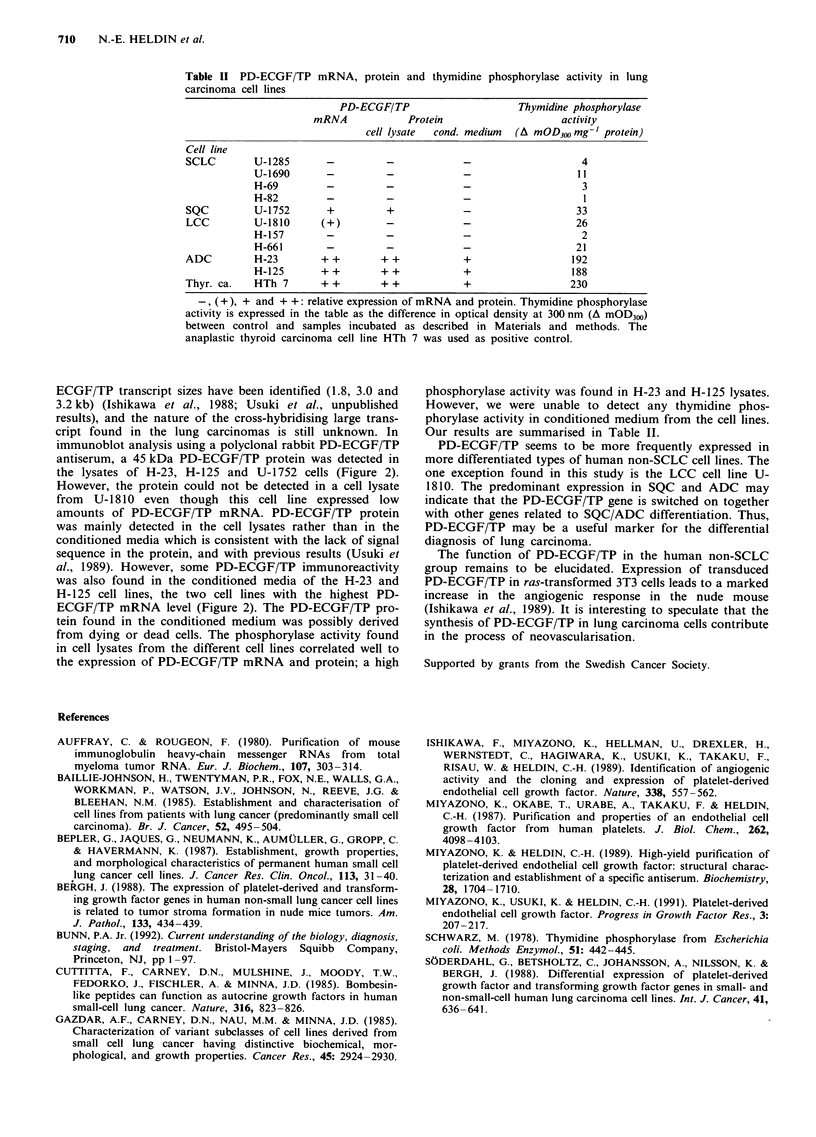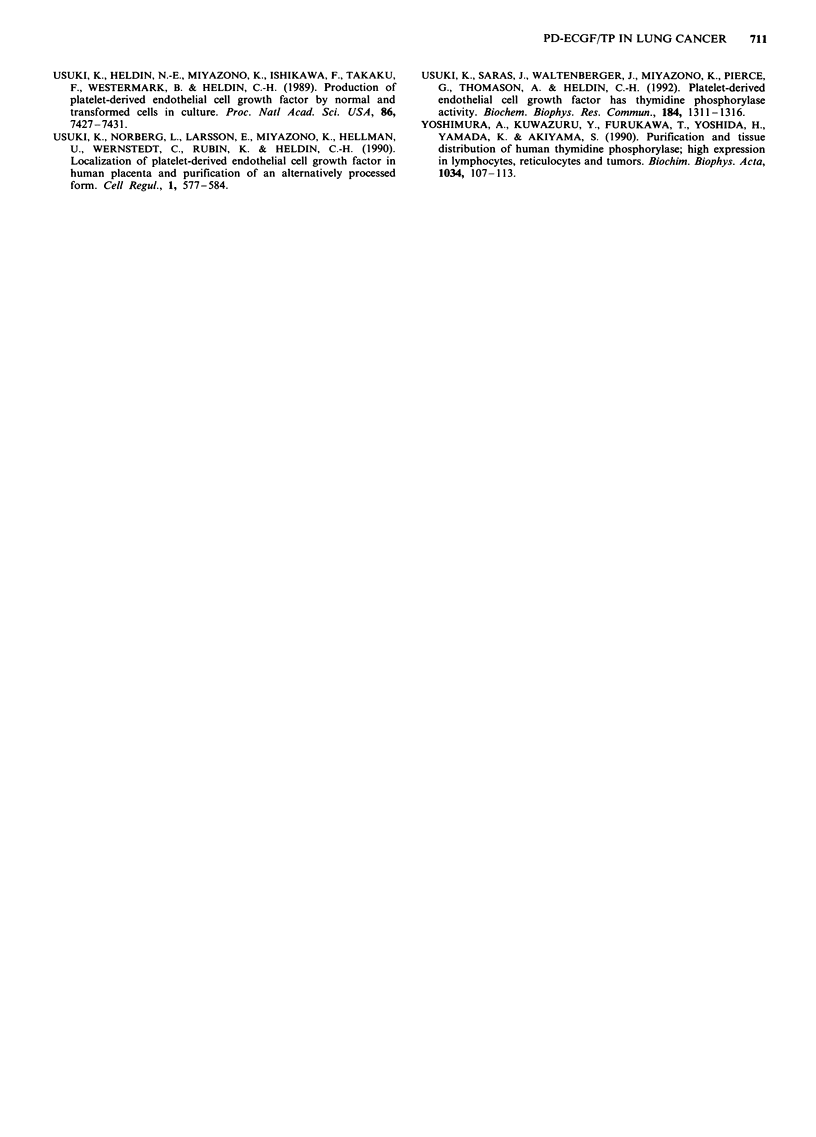# Differential expression of platelet-derived endothelial cell growth factor/thymidine phosphorylase in human lung carcinoma cell lines.

**DOI:** 10.1038/bjc.1993.414

**Published:** 1993-10

**Authors:** N. E. Heldin, K. Usuki, J. Bergh, B. Westermark, C. H. Heldin

**Affiliations:** Department of Pathology, University Hospital, Uppsala, Sweden.

## Abstract

**Images:**


Br. J. Cancer (1993), 68, 708-711
© Macmillan Press Ltd., 1993

### Differential expression of platelet-derived endothelial cell growth factor/thymidine phosphorylase in human lung carcinoma cell lines

N.-E. Heldin[1], K. Usuki[2], J. Bergh[3], B. Westermark[1] & C.-H. Heldin[2]

[1]*Department of Pathology and* [3]*Department of Oncology, University Hospital, S-751 85 Uppsala, Sweden;* [2]*Ludwig Institute for Cancer Research, Biomedical Centre, Box 595, S-751 23 Uppsala, Sweden.*

**Summary** In the present investigation we have studied the expression of platelet-derived endothelial cell growth factor/thymidine phosphorylase (PD-ECGF/TP) in ten different human lung carcinoma cell lines, four small cell carcinomas and six non-small cell carcinomas. None of the small-cell lung carcinoma cell lines demonstrated expression of PD-ECGF/TP mRNA. However, four of six of the non-small cell carcinoma cell lines expressed the 1.8 kb PD-ECGF/TP transcript. The cell lines derived from the single squamous cell carcinoma and the two adenocarcinomas expressed the PD-ECGF/TP mRNA, and were found to have the corresponding protein both in cell lysates and conditioned media as determined both by immunoblotting and measurement of thymidine phosphorylase activity. Only one of three studied large cell carcinoma cell lines expressed low levels of PD-ECGF/TP mRNA, but the corresponding PD-ECGF/TP protein was not demonstrated by immunoblotting.

Platelet-derived endothelial cell growth factor (PD-ECGF) was originally characterised as an angiogenesis factor (reviewed by Miyazono *et al.*, 1991); it was recently found to have 40% sequence similarity to thymidine phosphorylase of *Escherichia coli,* and to have thymidine phosphorylase activity (Usuki *et al.*, 1992). The amino acid sequence of platelet-derived endothelial cell growth factor/thymidine phosphorylase (PD-ECGF/TP), as deduced from a cDNA clone (Ishikawa *et al.*, 1989), revealed that PD-ECGF/TP lacks a signal sequence. PD-ECGF/TP has so far been demonstrated in platelets (Miyazono *et al.*, 1987), placenta (Usuki *et al.*, 1990), and in macrophage-like cells of lung and liver (Yoshimura *et al.*, 1990). Analysis of expression of PD-ECGF/TP protein and mRNA in cell lines revealed that certain epithelial cell lines were positive, including two out of three thyroid carcinoma cell lines (Usuki *et al.*, 1989).

Human lung cancer can be separated into two major categories, small cell lung cancer (SCLC) and non-SCLC (Bunn, 1992). The non-SCLC group can be subdivided into three morphological entities, squamous cell carincoma (SQC), adenocarcinoma (ADC) and large cell carcinoma (LCC) (Histological typing of lung tumours, WHO, Geneva 1981). The distinction between SCLC and non-SCLC is based on discriminative morphology, biochemical marker profile and clinical characteristics (Bunn, 1992). The SCLC group has high levels of a wide spectrum of neuroendocrine markers (Cuttitta *et al.*, 1985; Gazdar *et al.*, 1985; Baillie-Johnson *et al.*, 1985; Söderdahl *et al.*, 1988; Bepler *et al.*, 1987). The non-SCLC group expresses these neuroendocrine markers at considerably lower levels (Gazdar *et al.*, 1985; Baillie-Johnson *et al.*, 1985; Bunn, 1992). A heterogeneous mRNA expression for platelet-derived growth factor (PDGF) A- and B- chains, and transforming growth factor (TGF) -$\alpha$ and -$\beta$, have been demonstrated in six out of six studied human non-SCLC cell lines (Söderdahl *et al.*, 1988). The four studied SCLC cell lines were negative for the expression of PDGF and TGF-$\alpha$ or -$\beta$ (Söderdahl *et al.*, 1988). The non-SCLC cell lines have been demonstrated to induce a heterogeneous and collagen rich tumour stroma in nude mice xenografts from the non-SCLC cell lines while the SCLC cell lines were devoid of this capacity (Bergh, 1988). The stroma formation may relate to the production of PDGF and TGF-$\alpha$ or -$\beta$ or other yet unidentified growth factors with paracrine activity.

In the present work we report that PD-ECGF/TP is expressed in lung adeno- and squamous cell carcinoma cell lines, but not in the corresponding small cell carcinoma cell lines.

Correspondence: N.-E. Heldin, Department of Pathology, University Hospital, S-751 85 Uppsala, Sweden.
Received 8 January 1993; and in revised form 26 May 1993.

### Materials and methods

### *Cell culture*

The different lung carcinoma cell lines used in this study were the small-cell lung carcinomas (SCLC) U-1285, U-1690, H-69 and H-82, the large cell lung carcinomas (LCC) U-1810, H-157 and H-661, the lung adenocarcinomas (ADC) H-23 and H-125, and one squamous cell lung carcinoma (SQC) U-1752. The cells were grown in RPMI medium supplemented with 10% foetal calf serum (FCS) and the anaplastic thyroid carcinoma cell line was cultured in Eagle's minimum essential medium with 10% newborn calf serum (NCS). Antibiotics, 100 U of penicillin and 50 $\mu$g streptomycin ml$^{-1}$, were added to the medium. The SCLC cell lines were characterised by growth in clusters and the presence of different neuroendocrine markers according to Table I. The non-SCLC cell lines grow in monolayer and express epithelial markers and low levels of neuroendocrine markers (Table I).

### *Extraction of RNA*

Total RNA was extracted from cells using a lithium chloride/urea method described by Auffray and Rougeon (1980). Cells were homogenised in a solution (3 M lithium chloride, 6 M urea, 0.2% sodium dodecyl sulphate (SDS) and 1 $\mu$l ml$^{-1}$ of Antifoam A (Sigma Chemicals)) and left on ice overnight. The cell homogenate was centrifuged for 15 min at 16,000 *g* and the pellet was dissolved in a TES-buffer (10 mM

**Table I** Phenotypic properties of studied cell lines

| *Cell line* | *Diagnosis* | *NSE* | *EGF-rec. mRNA expression* | *In vitro growth* |
|---|---|---|---|---|
| *Small cell carcinoma cell line* | | | | |
| U-1285 | SCLC | 380 | ND | Clusters in suspension |
| U-1690 | SCLC | 2100 | – | Attached |
| H-69 | SCLC | 817 | – | Clusters in suspension |
| H-82 | SCLC | 317 | – | Clusters in suspension |
| *Non-small cell carcinoma cell lines* | | | | |
| U-1752 | SQC | <100 | + | Monolayer |
| U-1810 | LCC | <100 | + | Monolayer |
| H-157 | LCC | <100 | + | Monolayer |
| H-661 | LCC | ND | – | Monolayer |
| H-23 | ADC | <100 | + | Monolayer |
| H-125 | ADC | <100 | + | Monolayer |

NSE, Neuron-specific enolase in ng/mg protein; EGF-rec., epidermal growth factor receptor; ND, not determined.

triethanolamine pH 7.5, 1 mM EDTA, 0.5% SDS). After an extraction with phenol followed by an extraction with chloroform/isoamylalcohol (24:1), the total RNA was precipitated with 0.1 volumes of 3 M sodium acetate and 2.2 volumes of ethanol. The RNA was quantitated spectrophotometrically and subjected to a poly $(A)^+$ selection prior to the electrophoresis.

*Northern blot analysis*

Poly $(A)^+$ RNA samples (10 µg/lane) of the different lung carcinoma cell lines were size-fractionated by electrophoresis in formaldehyde-agarose and blotted onto a nitrocellulose filter. The filter was prehybridised for 4 h at 42°C in a buffer consisting of 20% formamide, 5 × SSC, 5 × Denhardt's solution, 5 mM $Na_2HPO_4$, 5 mM $NaH_2PO_4$, 0.5% SDS, and 200 µg salmon sperm per ml. Hybridisation was performed for 8 h at 42°C in the same buffer containing approximately $2 \times 10^6$ cpm of $^{32}$P-labelled PD-ECGF/TP cDNA probe (PL-5; 1.5 kb Eco R1 fragment; Ishikawa *et al.*, 1989) per ml. After the hybridisation period the filter was washed in 2 × SSC and 0.5% SDS at 60°C for 30 min. Autoradiography was performed at −70°C in the presence of intensifying screens (Du Pont).

*Immunoblot analysis*

Cell cultures were washed with medium and then incubated in serum-free medium for 24 h. The conditioned medium was collected and cells were solubilised in 0.15 M NaCl, 50 mM Tris-HCl pH 7.4, 1% Triton X-100, 1% deoxycholate, 0.1% SDS, 10 mM EDTA, 1 mM phenylmethylsulfonyl fluoride, 150 kallikrein inhibitor units aprotinin per ml, and centrifuged at 10,000 *g* for 20 min in order to clarify the samples. Samples were size fractionated by SDS-electrophoresis using gradient gels (10–18% acrylamide) and transferred to a nitrocellulose filter in a buffer consisting of 10% ethanol, 150 mM glycine, and 20 mM Tris-HCl pH 8.4, at 200 mA. In order to block the unspecific binding the filters were incubated in 0.15 M NaCl, 10 mM Tris-HCl pH 7.4, 5% bovine serum albumin. The filters were then incubated with specific PD-ECGF/TP rabbit antiserum (dilution 1:50) (Miyazono & Heldin, 1989), and washed twice in 0.15 M NaCl, 10 mM Tris-HCl pH 7.4, followed by two washes in 0.15 M NaCl, 10 mM Tris-HCl pH 7.4, 0.05% Triton X-100. The filters were then incubated for 30 min with swine anti-rabbit IgG diluted 1:1000 in 0.15 M NaCl, 10 mM Tris-HCl pH 7.4, and 0.1% bovine serum albumin. After washing as described above, the staining was performed with 100 mg $ml^{-1}$ nitrobluetetrazolium, 40 µg $ml^{-1}$ of 5-bromo-4-chloro-3-indolyl phosphate, 5 mM $MgCl_2$, 0.1 M ethanolamine buffer, pH 9.6.

*Thymidine phosphorylase assay*

Lysates were prepared from the cell lines by freeze-thawing cells 5 times in a buffer consisting of 50 mM Tris-HCl, pH 7.4, 0.15 M NaCl, and centrifuged for 15 min at 10,000 *g*. The cell lysates were incubated for 16 h at 37°C in 5 mM thymidine and 10 mM $K_3PO_4$, pH 7.4. At the end of incubation 0.3 ml of the reaction-mix was added to 0.7 ml of 0.5 M NaOH. The conversion of thymidine to thymine was measured spectrofotometrically at 300 nm (Schwartz, 1978). The thymidine phosphorylase activity found is expressed as the change in optical density at 300 nm ($\Delta$ $mOD_{300}$) per mg of protein in the cell lysates.

**Figure 1** Expression of PD-ECGF/TP mRNA. Poly $(A)^+$ RNA samples (10 µg/sample) were electrophoresed, blotted and hybridised with a PD-ECGF cDNA probe. A photograph of the resulting autoradiogram is shown. An anaplastic thyroid carcinoma cell line, HTh 7, was used as a positive control for the expression of PD-ECGF/TP mRNA (Usuki *et al.*, 1989).

**Figure 2** PD-ECGF/TP protein in ADC and SQC lung carcinomas. Conditioned media and lysates of the different lung carcinoma cell lines were subjected to immunoblotting, using a polyclonal PD-ECGF rabbit antiserum. Immunoglobulin complexes were visualised by alkaline phosphatase staining. Pure PD-ECGF/TP (100 ng) was used as control in the experiment. All lung carcinoma cell lines were analysed, but only the positive cell lines are shown in the figure.

### Results and discussion

Northern blot analysis of poly $(A)^+$ RNA extracted from the different cell lines showed an expression of PD-ECGF/TP transcripts in four of six of the non-SCLC cell lines (Figure 1). The positive cell lines were the two adenocarcinomas (H-23 and H-125), the squamous cell lung carcinoma (U-1752), as well as one of the three large cell lung carcinomas (U-1810). There was a marked difference in the amount of PD-ECGF/TP mRNA in the positive cell lines. As seen in Figure 1, the PD-ECGF/TP probe also hybridised to several mRNA's of different sizes in all studied cell lines except for the thyroid carcinoma cell line. So far three different PD-

**Table II** PD-ECGF/TP mRNA, protein and thymidine phosphorylase activity in lung carcinoma cell lines

| | | PD-ECGF/TP | | | Thymidine phosphorylase activity |
|---|---|---|---|---|---|
| | | mRNA | Protein | | |
| | | | cell lysate | cond. medium | ($\Delta$ $mOD_{300}$ $mg^{-1}$ protein) |
| Cell line | | | | | |
| SCLC | U-1285 | – | – | – | 4 |
| | U-1690 | – | – | – | 11 |
| | H-69 | – | – | – | 3 |
| | H-82 | – | – | – | 1 |
| SQC | U-1752 | + | + | – | 33 |
| LCC | U-1810 | (+) | – | – | 26 |
| | H-157 | – | – | – | 2 |
| | H-661 | – | – | – | 21 |
| ADC | H-23 | + + | + + | + | 192 |
| | H-125 | + + | + + | + | 188 |
| Thyr. ca. | HTh 7 | + + | + + | + | 230 |

–, (+), + and + +: relative expression of mRNA and protein. Thymidine phosphorylase activity is expressed in the table as the difference in optical density at 300 nm ($\Delta$ $mOD_{300}$) between control and samples incubated as described in Materials and methods. The anaplastic thyroid carcinoma cell line HTh 7 was used as positive control.

ECGF/TP transcript sizes have been identified (1.8, 3.0 and 3.2 kb) (Ishikawa *et al.*, 1988; Usuki *et al.*, unpublished results), and the nature of the cross-hybridising large transcript found in the lung carcinomas is still unknown. In immunoblot analysis using a polyclonal rabbit PD-ECGF/TP antiserum, a 45 kDa PD-ECGF/TP protein was detected in the lysates of H-23, H-125 and U-1752 cells (Figure 2). However, the protein could not be detected in a cell lysate from U-1810 even though this cell line expressed low amounts of PD-ECGF/TP mRNA. PD-ECGF/TP protein was mainly detected in the cell lysates rather than in the conditioned media which is consistent with the lack of signal sequence in the protein, and with previous results (Usuki *et al.*, 1989). However, some PD-ECGF/TP immunoreactivity was also found in the conditioned media of the H-23 and H-125 cell lines, the two cell lines with the highest PD-ECGF/TP mRNA level (Figure 2). The PD-ECGF/TP protein found in the conditioned medium was possibly derived from dying or dead cells. The phosphorylase activity found in cell lysates from the different cell lines correlated well to the expression of PD-ECGF/TP mRNA and protein; a high phosphorylase activity was found in H-23 and H-125 lysates. However, we were unable to detect any thymidine phosphorylase activity in conditioned medium from the cell lines. Our results are summarised in Table II.

PD-ECGF/TP seems to be more frequently expressed in more differentiated types of human non-SCLC cell lines. The one exception found in this study is the LCC cell line U-1810. The predominant expression in SQC and ADC may indicate that the PD-ECGF/TP gene is switched on together with other genes related to SQC/ADC differentiation. Thus, PD-ECGF/TP may be a useful marker for the differential diagnosis of lung carcinoma.

The function of PD-ECGF/TP in the human non-SCLC group remains to be elucidated. Expression of transduced PD-ECGF/TP in *ras*-transformed 3T3 cells leads to a marked increase in the angiogenic response in the nude mouse (Ishikawa *et al.*, 1989). It is interesting to speculate that the synthesis of PD-ECGF/TP in lung carcinoma cells contribute in the process of neovascularisation.

Supported by grants from the Swedish Cancer Society.

### References

AUFFRAY, C. & ROUGEON, F. (1980). Purification of mouse immunoglobulin heavy-chain messenger RNAs from total myeloma tumor RNA. *Eur. J. Biochem.*, **107**, 303–314.

BAILLIE-JOHNSON, H., TWENTYMAN, P.R., FOX, N.E., WALLS, G.A., WORKMAN, P., WATSON, J.V., JOHNSON, N., REEVE, J.G. & BLEEHAN, N.M. (1985). Establishment and characterisation of cell lines from patients with lung cancer (predominantly small cell carcinoma). *Br. J. Cancer*, **52**, 495–504.

BEPLER, G., JAQUES, G., NEUMANN, K., AUMÜLLER, G., GROPP, C. & HAVERMANN, K. (1987). Establishment, growth properties, and morphological characteristics of permanent human small cell lung cancer cell lines. *J. Cancer Res. Clin. Oncol.*, **113**, 31–40.

BERGH, J. (1988). The expression of platelet-derived and transforming growth factor genes in human non-small lung cancer cell lines is related to tumor stroma formation in nude mice tumors. *Am. J. Pathol.*, **133**, 434–439.

BUNN, P.A. Jr. (1992). *Current understanding of the biology, diagnosis, staging, and treatment.* Bristol-Mayers Squibb Company, Princeton, NJ, pp 1–97.

CUTTITTA, F., CARNEY, D.N., MULSHINE, J., MOODY, T.W., FEDORKO, J., FISCHLER, A. & MINNA, J.D. (1985). Bombesin-like peptides can function as autocrine growth factors in human small-cell lung cancer. *Nature*, **316**, 823–826.

GAZDAR, A.F., CARNEY, D.N., NAU, M.M. & MINNA, J.D. (1985). Characterization of variant subclasses of cell lines derived from small cell lung cancer having distinctive biochemical, morphological, and growth properties. *Cancer Res.*, **45**: 2924–2930.

ISHIKAWA, F., MIYAZONO, K., HELLMAN, U., DREXLER, H., WERNSTEDT, C., HAGIWARA, K., USUKI, K., TAKAKU, F., RISAU, W. & HELDIN, C.-H. (1989). Identification of angiogenic activity and the cloning and expression of platelet-derived endothelial cell growth factor. *Nature*, **338**, 557–562.

MIYAZONO, K., OKABE, T., URABE, A., TAKAKU, F. & HELDIN, C.-H. (1987). Purification and properties of an endothelial cell growth factor from human platelets. *J. Biol. Chem.*, **262**, 4098–4103.

MIYAZONO, K. & HELDIN, C.-H. (1989). High-yield purification of platelet-derived endothelial cell growth factor: structural characterization and establishment of a specific antiserum. *Biochemistry*, **28**, 1704–1710.

MIYAZONO, K., USUKI, K. & HELDIN, C.-H. (1991). Platelet-derived endothelial cell growth factor. *Progress in Growth Factor Res.*, **3**: 207–217.

SCHWARZ, M. (1978). Thymidine phosphorylase from *Escherichia coli. Methods Enzymol.*, **51**: 442–445.

SÖDERDAHL, G., BETSHOLTZ, C., JOHANSSON, A., NILSSON, K. & BERGH, J. (1988). Differential expression of platelet-derived growth factor and transforming growth factor genes in small- and non-small-cell human lung carcinoma cell lines. *Int. J. Cancer*, **41**, 636–641.

USUKI, K., HELDIN, N.-E., MIYAZONO, K., ISHIKAWA, F., TAKAKU, F., WESTERMARK, B. & HELDIN, C.-H. (1989). Production of platelet-derived endothelial cell growth factor by normal and transformed cells in culture. *Proc. Natl Acad. Sci. USA*, **86**, 7427–7431.

USUKI, K., NORBERG, L., LARSSON, E., MIYAZONO, K., HELLMAN, U., WERNSTEDT, C., RUBIN, K. & HELDIN, C.-H. (1990). Localization of platelet-derived endothelial cell growth factor in human placenta and purification of an alternatively processed form. *Cell Regul.*, **1**, 577–584.

USUKI, K., SARAS, J., WALTENBERGER, J., MIYAZONO, K., PIERCE, G., THOMASON, A. & HELDIN, C.-H. (1992). Platelet-derived endothelial cell growth factor has thymidine phosphorylase activity. *Biochem. Biophys. Res. Commun.*, **184**, 1311–1316.

YOSHIMURA, A., KUWAZURU, Y., FURUKAWA, T., YOSHIDA, H., YAMADA, K. & AKIYAMA, S. (1990). Purification and tissue distribution of human thymidine phosphorylase; high expression in lymphocytes, reticulocytes and tumors. *Biochim. Biophys. Acta*, **1034**, 107–113.